# Social thinking is for doing: the posterior cerebellum supports predictions of social actions based on personality traits

**DOI:** 10.1093/scan/nsab087

**Published:** 2021-07-14

**Authors:** Naem Haihambo, Qianying Ma, Chris Baeken, Natacha Deroost, Kris Baetens, Elien Heleven, Frank Van Overwalle

**Affiliations:** Department of Psychology, Vrije Universiteit Brussel, Brussels 1050, Belgium; Center for Neuroscience, Vrije Universiteit Brussel, Brussels 1050, Belgium; Department of Psychology, Vrije Universiteit Brussel, Brussels 1050, Belgium; Center for Neuroscience, Vrije Universiteit Brussel, Brussels 1050, Belgium; Department of Psychology, Vrije Universiteit Brussel, Brussels 1050, Belgium; Center for Neuroscience, Vrije Universiteit Brussel, Brussels 1050, Belgium; Department of Psychology, Vrije Universiteit Brussel, Brussels 1050, Belgium; Center for Neuroscience, Vrije Universiteit Brussel, Brussels 1050, Belgium; Department of Psychology, Vrije Universiteit Brussel, Brussels 1050, Belgium; Center for Neuroscience, Vrije Universiteit Brussel, Brussels 1050, Belgium; Department of Psychology, Vrije Universiteit Brussel, Brussels 1050, Belgium; Center for Neuroscience, Vrije Universiteit Brussel, Brussels 1050, Belgium; Department of Psychology, Vrije Universiteit Brussel, Brussels 1050, Belgium; Center for Neuroscience, Vrije Universiteit Brussel, Brussels 1050, Belgium

**Keywords:** posterior cerebellum, action prediction, personality traits, social action sequencing

## Abstract

Can we predict the future by reading others’ minds? This study explores whether attributing others’ personality traits facilitates predictions about their future actions and the temporal order of these future actions. Prior evidence demonstrated that the posterior cerebellar crus is involved in identifying the temporal sequence of social actions and the person’s traits they imply. Based on this, we hypothesized that this area might also be recruited in the reverse process; that is, knowledge of another person’s personality traits supports predictions of temporal sequences of others’ actions. In this study, participants were informed about the trait of a person and then had to select actions that were consistent with this information and arrange them in the most likely temporal order. As hypothesized, the posterior cerebellar crus 1 and crus 2 were strongly activated when compared to a control task which involved only the selection of actions (without temporal ordering) or which depicted non-social objects and their characteristics. Our findings highlight the important function of the posterior cerebellar crus in the prediction of social action sequences in social understanding.

## Highlights

- Previous research has demonstrated the role of the cerebellar crus in inferring traits from social action sequences.
- We investigated the reverse logic: Is the posterior cerebellar crus also recruited when people make predictions on novel action sequences based on trait information?
- The posterior cerebellar crus (1 and 2) and lobule IX are preferentially involved in predicting social action sequences from trait information.
- Classic mentalizing cortical areas, specifically the temporoparietal junction and medial prefrontal cortex, are activated alongside the cerebellum in predicting social action sequences.

## Introduction

As human beings, we navigate through our social environments by reading the minds of others and anticipate future interactions based on this information. This dynamic ability to attribute mental states to others is called social mentalizing and involves reading mental states such as desires, intentions and personality traits in social contexts (Van Overwalle, 2009; Schurz *et al.*, 2014; Molenberghs *et al.*, 2016). This mentalizing ability allows us to use these mental state inferences as a basis to make reliable predictions about future behaviors of others and outcomes of interactions, so that we can anticipate future social encounters to our (mutual) benefit, which is arguably the ultimate goal of social behaviors (Frith and Frith, 2006; Pisotta and Molinari, 2014; Molinari and Masciullo, 2019), and subsequently plan our actions and responses accordingly. A helpful way to predict how people might behave is by knowing their personality traits (Hassabis *et al.*, 2014). To illustrate, when learning from others that our new neighbor is friendly, we would predict that she will behave in a friendly manner when we meet her for the first time and, by extension, register when she is acting in ways that are inconsistent with this trait information (e.g. not saying ‘hallo’ when passing her on the street).

Social mentalizing and the brain neuroscientific research have contributed significantly to understanding the neural underpinnings of social mentalizing processes. This exploration has focused primarily on the cerebral cortex and, in particular, the mentalizing network (see meta-analyses Van Overwalle, 2009; Van Overwalle and Baetens, 2009; Schurz *et al.*, 2014), which includes key areas such as the temporoparietal junction (TPJ), medial precuneus and medial prefrontal cortex (mPFC). However, recently the focus of mentalizing research has been extended to the cerebellum. A large-scale seminal meta-analysis by Van Overwalle *et al.* (2014), which included over 350 functional magnetic resonance imaging (fMRI) studies involving a large variety of social cognitive tasks, revealed a robust activation of the cerebellum in social cognition tasks in over one-third of studies included across the majority of tasks. These findings were further reinforced by fMRI studies demonstrating that there was a distinct functional connectivity between the posterior cerebellar crus and cortical mentalizing areas during social mentalizing (Van Overwalle *et al.*, 2019c, 2020c). Furthermore, fMRI studies have shown that higher-order mentalizing, involving abstractions such as trait inferences (Baetens *et al.*, 2014; Pu *et al.*, 2020), higher-order belief inferences (Lewis *et al.*, 2017) and imagining past and future events (Van Hoeck *et al.*, 2013) strongly activate cerebellar areas (Van Overwalle *et al.*, 2019a, 2020c). The role of the cerebellum has been confirmed by Yeo *et al.* (2011), who identified a distinct ‘default mode’ network in the cerebrum encompassing social mentalizing (Andrews-Hanna *et al.*, 2010), which extends to the posterior cerebellum (Buckner *et al.*, 2011).

However, what is the function of the cerebellum in social cognition? The primary function of the cerebellum involves automatizing motor and non-motor processes by creating internal and adaptive models of movement and mental sequences that allow prediction and spontaneous execution of these movements and mental thoughts and their anticipated consequences (e.g. learning to ride a bicycle or playing the piano; Leggio and Molinari, 2015), which allows agents to free their mind to focus on novel and challenging information in the environment and the future. Hence, ‘cerebellar activity is related more to the expectancy of future events than with the registration of ongoing activities’ (Molinari *et al.*, 2009, p. 399). Although cerebellar research initially focused on motor behavior, during the last decade, increasing evidence has pointed to cerebellar involvement in mental processes (Ito, 2008; Pisotta and Molinari, 2014), such as social mentalizing (Van Overwalle *et al.*, 2019a, 2020b, 2020c); thus, suggesting that social mentalizing and behavior often requires coordination of a number of social cognitive elements in the correct sequence, exactly as with motor coordination. In the present manuscript, we will investigate the hypothesis proposed by Molinari *et al.* (2009) that the cerebellum supports predictions of future actions, and more in particular, in the domain of social mentalizing: Does the cerebellum support the prediction of social actions based on prior mental inferences?

### The sequencing role of the cerebellum

To investigate the role of the cerebellum in social mentalizing, recent studies using fMRI (Heleven *et al.*, 2019) and cerebellar patients (Van Overwalle *et al.*, 2019a) have used mentalizing tasks in which sequences of social actions play a critical role. A typical example is the picture sequencing task, in which cartoon-like drawings depicting various social actions were presented in a random order, and participants were instructed to put these pictures in the correct chronological order (Langdon and Coltheart, 1999). To put some of the stories in the correct order, participants must understand the beliefs of the protagonist. Studies with cerebellar patients (Van Overwalle *et al.*, 2019a) and neuroimaging studies on healthy participants (Heleven *et al.*, 2019) demonstrated that the posterior cerebellar crus was activated for stories involving mentalizing about the other person’s beliefs in comparison with routine social scripts or non-social mechanical stories.

Perhaps more critical for the present research, in a recent study by Pu *et al.* (2020), participants were asked to remember the temporal order of social action sequences that implied the same trait of the agent, and the results revealed that the posterior cerebellar crus was recruited in comparison with social events without sequencing instruction and non-social sequencing. Although prior studies have also investigated imagination about future events (for review, see Hassabis and Maguire, 2009), these studies did not include trait information nor did they ask participants to generate sequences of future social actions (Addis *et al.*, 2007; Hassabis *et al.*, 2007) and did not reveal activation of the cerebellum. Thus, it seems that sequencing of social actions is critical in activating the cerebellar crus when anticipating future actions.

### Present study

To address the prediction of social actions and their temporal order based on mentalizing inferences, we reversed the task logic of Pu *et al.* (2020), where participations had to memorize a given order of social actions and then had to infer the trait of the protagonist. Specifically, in our study, participants were first given the trait of a protagonist (e.g. Fumak is dishonest). Afterwards, they had to select four out of six possible actions that would depict actions consistent with the trait presented and put them in the correct chronological order. To verify that sequencing and social mentalizing were important components of posterior cerebellar activation, we build in several control conditions involving a selection-only task that did not involve generating a sequence and a non-social task (i.e. with and without generating a sequence) involving objects and their characteristics (e.g. a light feather).

Our novel hypothesis is that the posterior cerebellar crus is involved in predicting the most likely social actions in their correct temporal order, based on the trait information about the protagonist given earlier. In particular, we expect stronger activation of the posterior cerebellar crus in the social sequencing task where the correct order of predicted actions needs to be generated, compared to the selection-only and the non-social control tasks. Moreover, given the close connectivity between the posterior cerebellum and cortical mentalizing areas (Van Overwalle *et al.*, 2017, 2019a, 2020a), and because trait information needs to be processed to make adequate predictions about future trait-related actions, we also predict that along with concurrent posterior cerebellar activation, activation in the TPJ and mPFC, two key cortical mentalizing areas involved in trait attribution, will also be present.

## Method

### Participants

There were 27 (eight males) healthy, right-handed, Dutch-speaking participants (mean age 24 years, s.d. = 3 years). They all had normal or corrected-to-normal vision and reported no neurological or psychiatric disorders. No participants were excluded due to excessive head movement (outlier scans > 5%). Informed consent was obtained with the approval of the Medical Ethics Committee at the Gent University Hospital, where the study was conducted. Participants were paid 20 euros in exchange for their participation, and additional transportation costs to the experiment site were reimbursed.

### Stimulus material

**Fig. 1.**
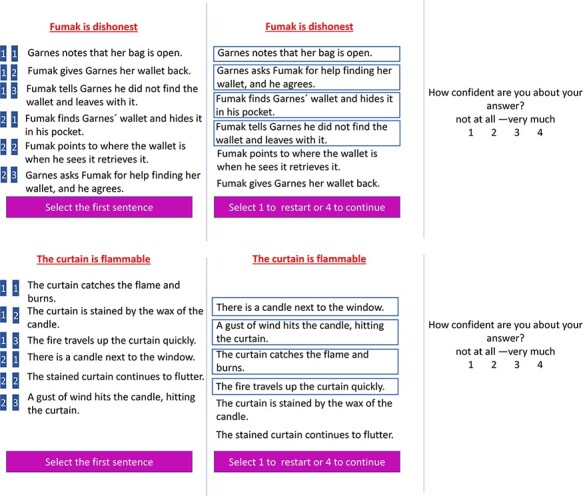
Illustration of a trial from the social sequencing (top panel) and non-social sequencing (bottom panel) conditions. Left panel: Participants were presented six action sentences (randomly ordered) and were required to select the four sentences that fit best with the person trait/object feature and to order them in the correct order (ignoring the trait-inconsistent sentences) using two consecutive button presses on a four-button response box (with responses indicated on a blue background). Middle panel: The ordering as chosen by a participant (the selected four sentences were ordered from top to bottom and marked by squares surrounding them). Right panel: After this, participants were asked to rate their confidence on a 4-point confidence rating scale.

A novel trait-implying action sequence prediction task was developed (Figure 1). In this task, participants were presented with a fictitious main agent and his/her trait (e.g. Fumak is dishonest), along with six socially interactive trait-implying sentences. We used fictional Star-Trek-like names, to avoid using names of persons that were familiar to the participants. Participants had to infer what the agent would do based on the trait information provided.

In the social condition, each trial consisted of a set of six trait-implying sentences describing interactions between two agents (the target agent whose trait was presented and another agent). Of these six action sentences, two were neutral, two consistent and two inconsistent with respect to the target person’s trait (Figure 1). In the non-social control condition, participants were presented with an object and its characteristic (e.g. the curtain is flammable) along with six sentences, of which two were neutral, two consistent and two inconsistent with respect to the object’s characteristic. Across these two conditions, the sentence sets were generally identical, with the exception that the social conditions included social agents performing social actions, whereas the non-social conditions included non-social objects in a cause-and-effect scenario with the environment. All sentences were newly developed for this study, although they were inspired by the previous trait-implying task by Pu *et al.* (2020).

All sentences in the social and non-social condition sets were tested to have the sequence that was built in. In a pilot study, 36 additional participants who did not participate in the fMRI study received the same task described above and were requested to order the sentences in each story set. Story sets were included for the present study when the built-in sequencing was identified above average or 65%. This level was chosen to allow enough variation in difficulty of and performance at the task. The story sets were randomly distributed between the sequencing and selection-only conditions.

### Procedure

Participants were informed that there would be two tasks in the experiment: sequencing and selection only.

In the sequencing task (see Figure 1), on each trial, participants were first shown a trait of one agent (social condition) or a characteristic of one object (non-social condition). The agents and their given trait (or the objects and their characteristic) appeared in red on the top of the screen and remained there for the duration of that trial. After 1000 ms, six sentences were shown on the screen one-by-one for 1300 ms each, in a random order for each participant and for each trial. The timing for reading each sentence was determined by a stimulus materials pilot study, indicating that the shortest time needed to read the sentences was approximately 1300 ms. After reading, all sentences were shown together on screen.

In the sequencing task, participants were instructed to ‘select only the four sentences that fit the trait of the person or characteristic of the object and put them in the correct chronological order’. In the selection-only task, however, participants were told that ‘the sentences are now already put in the correct order’ and that they only had to ‘select the four sentences that fit the trait of the person or characteristic of the object’ without generating the correct order. They were further told to execute ‘this task correctly and as quickly as possible. Your time is measured from the presentation of the event until you indicate that you are ready’. Participants selected the sentences using two consecutive button presses on a four-button response box with their left hand. This was practiced outside the scanner so that participants were familiar with this response procedure before scanning. To continue or cancel (i.e. redo the ordering of the sentences), they had to press a button at the end of that trial, by selecting button ‘1 to restart or 4 to continue’. Because metacognitive confidence may effect brain activation during responding, at the end of the trial, participants were asked ‘How confident are you about your answer?’ and they answered with a button press on the response box using a 4-point scale ranging from 1 = ‘not at all’ to 4 = ‘very much’. All trials and confidence ratings were preceded by a blank screen with a fixation cross, jittered randomly between 1 and 2 s.

In the selection-only task the procedure was identical to the sequencing task (see Figure 1), but the sentences were different although drawn from the same piloting pool. Another exception was that the sentences were not presented in a random order, but in their correct order; that is, with a pair of trait-neutral sentences presented first and a pair of consistent and a pair of inconsistent sentences presented together in the middle or last position (randomly determined for each trial). Thus, while in the sequencing task, inconsistent sentences could appear at all positions 1–6, while in the selection-only task, they appeared only at positions 3–6. Participants had to only select one pair of two sentences out of the two consistent and inconsistent pairs that fitted the person trait or object characteristic. Sentences were presented for 1100 instead of 1300 ms, because participants needed less time, as they did not need to plan the order of sentences. This timing was based on a previous behavioral pilot study of the same task.

Before entering the scanner, and during three practice trials in the scanner, participants read the instructions and practiced the response presses, using sentences that were not part of the fMRI experiment, to make sure they understood how to make a selection and order the sentences. The whole experiment out and in the scanner was presented in E-Prime 2.0 (https://www.pstnet.com/eprime; Psychology Software Tools), running on a Windows 10 and Windows XP computer, respectively.

In total, participants completed 44 trials, each consisting of six sentences that differed across all conditions. Each sequencing or selection-only task consisted of 11 social and 11 non-social trials, or 22 trials in total per task. Participants first received the sequencing task, after which they received the selection-only task. This was done so that participants were not primed with the correct structure of already ordered sentences in the sequencing task. For each task, the social and non-social trials (i.e. sentence sets) were presented in a random order for each participant.

### Questionnaires

The Dutch version of the Autism Questionnaire (AQ; Hoekstra *et. al.*, 2008) was administered to detect any individual with high AQ scores. The AQ, originally developed in English (Baron-Cohen *et. al*., 2001), is a 50-item questionnaire assessing autism symptoms in adults of average intelligence. Responses on each item require participants to indicate agreement or disagreement on a 4-point scale, ranging from ‘definitely agree’, ‘slightly agree’, ‘slightly disagree’ to ‘definitely disagree’. With the exception of one participant, all participants completed the AQ. Mean score was 14.54, ranging between 7 and 29, well below the clinical threshold of 32.

### Imaging procedure and pre-processing

Images were collected with a Siemens Magnetom Prisma fit scanner system (Siemens Medical Systems, Erlangen, Germany) using a 64-channel radiofrequency head coil. Stimuli were projected onto a screen at the end of the magnet bore that participants viewed by way of a mirror mounted on the head coil. Participants were placed headfirst and supine in the scanner bore and were instructed not to move their heads to avoid motion artifacts. Foam cushions were placed within the head coil to minimize head movements. First, high-resolution anatomical images were acquired using a T1-weighted 3D magnetization-prepared rapid gradient-echo (MPRAGE) sequence (Repetition Time (TR) = 2250 ms, Echo Time (TE) = 4.18 ms, Inversion Time (TI) = 900 ms, Field of View (FOV) = 256 mm, flip angle = 9º, voxel size = 1 × 1 × 1 mm). Second, a fieldmap was calculated to correct for inhomogeneities in the magnetic field (Cusack and Papadakis, 2002). Third, whole-brain functional images were collected in a single run using a T2*-weighted gradient echo sequence, sensitive to Blood Oxygenation Level Dependant (BOLD) contrast (TR = 1000 ms, TE = 31.0 ms, FOV = 210 mm, flip angle = 52º, slice thickness = 2.5 mm, distance factor = 0%, voxel size = 2.5 × 2.5 × 2.5 mm, 56 axial slices, acceleration factor GeneRalized Autocalibrating Partial Parallel Acquisition (GRAPPA) = 4).

SPM12 (Wellcome Department of Cognitive Neurology, London, UK) was used to process and analyze the fMRI data. To remove sources of noise and artifact, data were preprocessed. Functional data were corrected for differences in acquisition time between slices for each whole-brain volume, realigned to correct for head movement and co-registered with each participant’s anatomical data. Then, the functional data were transformed into a standard anatomical space (2 mm isotropic voxels) based on the ICBM152 brain template (Montreal Neurological Institute, MNI). Normalized data were then spatially smoothed (6 mm full width at half-maximum) using a Gaussian Kernel. Finally, using the Artifact Detection Tool (http://web.mit.edu/swg/art/art.pdf; http://www.nitrc.org/projects/artifact_detect), the pre-processed data were examined for excessive motion artifacts and for correlations between motion and experimental design, and between global mean signal and experimental design. Outliers were identified in the temporal differences series by assessing between-scan differences (*Z*-threshold: 3.0 mm; scan-to-scan movement threshold: 0.5 mm; rotation threshold: 0.02 radians). These outliers were omitted from the analysis by including a single regressor for each outlier. A default high-pass filter was used of 128s and serial correlations were accounted for by the default auto-regressive model.

### Statistical analysis of behavioral data

Accuracy for the sequencing task was calculated in the same manner as in the false belief picture sequencing task by Heleven *et al.* (2019), based on the original scoring system by Langdon and Coltheart (1999), where the correct selection of the first and final sentences garnered 2 points each, while the correct selection of the second and third sentences garnered 1 point each, with a maximum score of 6. The rationale for this scoring system is that identification of the first and last sentences is most difficult, while identification of intermediate sentences is relatively easy (Langdon and Coltheart, 1999). Accuracy for the selection-only task was calculated by giving 1 point for a correct selection and 0 points for an incorrect selection. The response time (RT) was calculated by timing the whole trial, after all six sentences were all presented on screen for the first time and the prompt to select or sequence the sentences appeared.

A repeated measure analysis of variance (ANOVA) with domain (social *vs* non-social) and task (sequencing *vs* selection only) within-participants factors was conducted on accuracy and RT using ISM SPSS Statistics 26 software. The alpha level for pairwise comparisons was set at 0.05 and was reported when significant interactions were revealed.

### Statistical analysis of neuroimaging data

At the first (single participant) level, for each task, the event-related design was modeled for each condition (i.e. social sequencing, non-social sequencing control, social selection-only control and non-social selection-only control). The onset of each trial was set after all six sentences were presented for the first time, and the prompt to select or sequence the sentences appeared. Based on considerations of how response processes might have evolved during a trial and our aim to select equivalent timings for fMRI analysis across conditions, duration was set from the onset of the trial till the time participants selected the first trait-consistent sentence. This timing reflects the same process across the two Tasks (sequencing and selection only). Specifically, we took the time from the onset when the sentences were presented and (in the sequencing condition) the two initial trait-neutral sentences had to be selected or (in the selection-only condition) were already given, till the selection of the first trait-consistent sentence. All trials were analyzed, irrespective of whether selection or sequencing was correct, because we assumed that participants’ selection and sequencing of the first trait-consistent sentence was based on what they believed to be correct. When a trial was canceled and redone, analysis was performed on the final sentence selection.

At the second (group) level, whole-brain random effects analysis using one-way within-participants ANOVA was used, contrasting all conditions with each other, with the most critical contrasts being social sequencing > social selection only and social sequencing > non-social sequencing to test the role of sequencing and the social domain, respectively, in activating cerebellar crus. Significance was set at the cluster-defining uncorrected threshold of *P *< 0.001, followed by a cluster-wise familywise error (FWE)-corrected threshold *P* < 0.05, with a minimum cluster extent of 10 voxels. We also tested our hypotheses more directly by performing a region of interest (ROI) analysis, using spheres centered on a priori MNI coordinates for the cerebellar crus 1 and crus 2 (±40 −70 −40 and ±24 −76 −40, respectively; Van Overwalle *et al.*, 2020a) and a 15-mm radius. ROI analyses were done using a small volume (rather than the whole-brain volume) for multiple comparison correction with the same thresholds as the whole-brain analysis. To avoid redundancy, however, we reported ROI results only when whole-brain contrasts were not significant (i.e. denoted by ‘ROI’ in tables). In addition to this, a parametric regression analysis was conducted at the second level, investigating whether brain activity covaried with mean confidence ratings in each condition. Due to a lack of variation in responses in some conditions, only 17 participants were included in the parametric analysis.

## Results

### Behavioral results

#### Accuracy

For accuracy, the results revealed no significant main effects of domain or task, but their interaction was significant [*F*(1, 26) = 10.54, mean squared error (MSE)* = *0.006*, P* < 0.01, η_p_^2^ = 0.29]. Further, simple effect analyses revealed significant differences between social and non-social domain in the selection-only task. Accuracy in the non-social condition was significantly higher than the social condition [social: *M* = 89%, s.d. = 16%; non-social: *M* = 96%, s.d. = 6%; *F*(1, 26) = 18.36, *P* < 0.001, η_p_^2^ = 0.28, MD = 0.41]. For the sequencing task, accuracy was generally high for both domains (social: *M* = 90%, s.d. = 13%; non-social: *M* = 86%, s.d. = 11%) with no significant differences (*P* > 0.05).

#### RT

For RT, the results revealed no significant main effect for domain, but showed significant main effects for task [*F*(1, 26) = 573.98, MSE* = *64 878*, P* < 0.001, η_p_^2^ = 0.96], as well a significant interaction [*F*(1, 26) = 9.01, MSE* = *131, *P* < 0.01, η_p_^2^ = 0.26]. Further, simple analyses revealed significant differences between sequencing and selection-only task in the social domain [*F*(1, 26) = 557.79, *P* < 0.001, η_p_^2^ = 0.96, MD = 46.82] as well as in the non-social domain between sequencing and selection-only tasks [*F*(1, 26) = 475.24, *P* < 0.001, η_p_^2^* *= 0.95, MD = 51.22]. For the selection-only task, RT was slower in the social domain compared to the non-social domain (social: *M =* 9 s, s.d. = 5 s; non-social: *M =* 6 s, s.d.* =* 4 s). In the sequencing task, no significant difference was found between domains (social: *M *= 56 s, s.d. = 12 s; non-social: *M *= 57 s, s.d. = 15 s; *P* > 0.05).

Taken together, participants performed both faster and more accurately on the non-social selection-only task, compared to the social selection-only task.

#### Correlation of confidence with accuracy and RT

We used a Spearman’s correlation to test the relationship between average confidence ratings and participant performance. In the sequencing task, significant positive correlations were found between confidence ratings and accuracy in the two domains (social: *r *= 0.57, *P *< 0.01; non-social: *r *= 0.52, *P *< 0.01). No other significant correlations were found for RTs or for the selection-only task (*P* > 0.10).

### fMRI results

#### Social sequencing vs non-social sequencing

**Fig. 2.**
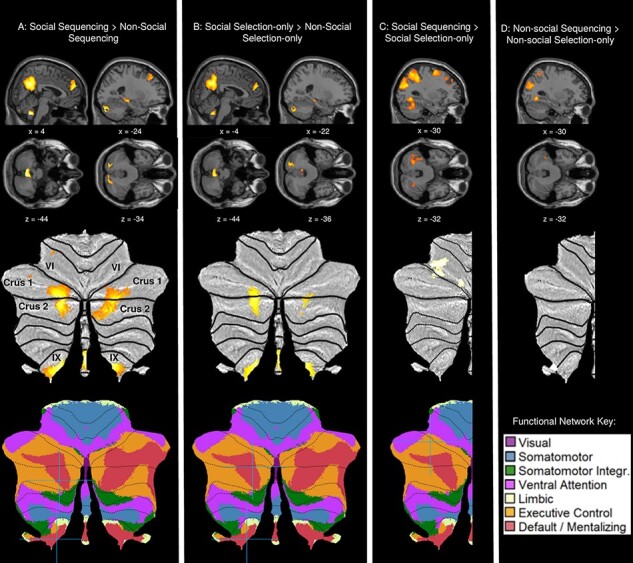
Sagittal and transverse views of the experimental contrasts involving sequencing and selection-only conditions, visualized at a whole-brain uncorrected threshold of *P* < 0.001, together with visualization on SUIT flatmaps of the cerebellum (with labeling of cerebellar lobules in panel (A). (A) Social sequencing > non-social sequencing contrast and (B) social selection-only > non-social selection-only contrast, both showing significant clusters (*P* < 0.05, FWE corrected) in cerebellar crus 1 and crus 2, as well as cerebellar lobule IX. (C) Social sequencing > social selection-only contrast showing activation in cerebellar crus 1 using a small volume correction. (D) Non-social sequencing > non-social selection-only contrast, showing no cerebellar activation. Peak activations of significant contrasts are also indicated with a blue crosshair on functional network flatmaps from Buckner *et al.* (2011; http://www.diedrichsenlab.org/imaging/AtlasViewer/viewer.html).

To test the hypothesis that the posterior cerebellar crus is preferentially involved in the sequencing of social rather than non-social actions, we computed a social sequencing > non-social sequencing contrast (Figure 2A; Table 1). As expected, this contrast revealed significant activation of the posterior cerebellum, predominantly crus 1 and crus 2, and in the TPJ (i.e. angular gyrus) and mPFC (i.e. superior medial gyrus), which are key cortical mentalizing areas. Unexpectedly, the cerebellar lobule IX was also robustly activated in this contrast. In addition, (sub)cortical areas were activated in the middle temporal frontal gyrus and the middle frontal gyrus including the anterior cingulate cortex (ACC), the superior frontal gyrus and the hippocampus. The opposite contrast (non-social sequencing > social sequencing) did not reveal activation in any of the areas of interest (i.e. crus, TPJ and mPFC).

**Table 1.** Whole-brain analysis comparing social *vs* non-social sequencing

Brain label/contrast	MNI coordinates			Voxels	*T*
	*x*	*y*	*z*		
Social sequencing > non-social sequencing					
Right cerebellum (IX)	4	−54	−44	277	7.65***
Left cerebellum (crus 2)	−24	−82	−34	231	6.56***
Left cerebellum (crus 2)	−14	−84	−32		3.950
Right cerebellum (crus 1)	28	−80	−32	199	5.74**
Right cerebellum (crus 2)	18	−84	−34		5.41*
Right cerebellum (crus 2)	10	−82	−38		4.630
Right precuneus	8	−58	30	2477	7.86***
Left precuneus	0	−62	32		7.23***
Left middle cingulate cortex	−4	−48	34		6.99***
Right middle temporal gyrus	54	−56	20	1001	7.51***
Right supramarginal gyrus	54	−46	24		7.15***
Right angular gyrus	44	−62	36		4.130
Left angular gyrus, including TPJ	−40	−56	26	1527	7.43***
Left angular gyrus	−54	−64	24		7.16***
Left angular gyrus	−42	−68	36		4.95*
	46	−34	−2		5.44*
Right middle temporal gyrus	54	−38	0	122	3.990
	−20	−16	−10		4.97*
Left hippocampus	−24	−28	−10	162	4.69
Left middle frontal gyrus	−28	28	52	211	4.71
Left superior frontal gyrus	−24	34	46		4.410
Left middle frontal gyrus	−28	18	48		4.05
Left superior medial gyrus	0	54	26	785	6.06**
Right ACC, including mPFC	6	50	20		5.73**
Left superior medial gyrus	−6	54	38		5.55*
Social sequencing < non-social sequencing					
Left supramarginal gyrus	−62	−34	30	423	5.37*

Coordinates refer to the MNI stereotaxic space. Significance for the whole-brain analysis was set at the cluster-defining uncorrected threshold of *P* < 0.001 and the cluster-wise FWE-corrected threshold of *P* < 0.05, with voxel extent ≥ 10.

* *P* < 0.05,

** *P* < 0.01,

*** *P* < 0.001 (peak FWE corrected).

#### Social selection- only vs non-social selection only

To further explore whether the preferential involvement of social material is limited to sequencing and not selection, we ran the same contrast for the selection-only conditions, that is, social selection only > non-social selection only (Figure 2B; Table 2). Similar activations were observed in the cerebellar crus 2 and lobule IX, but not in crus 1 like in the sequencing task. As hypothesized, activation was also observed in the cortical TPJ (i.e. angular gyrus) and mPFC (i.e. superior medial gyrus). Further (sub)cortical activations were observed in the precuneus, supramarginal gyrus, as well as the superior frontal and temporal gyrus. The opposite contrast (non-social selection only > social selection only) did not reveal activation in areas of interest (i.e. crus, TPJ and mPFC), revealing activations only in the supramarginal gyrus.

**Table 2.** Whole-brain analysis comparing social *vs* non-social selection only

	MNI coordinates				
Brain label/contrast	*x*	*y*	*z*	Voxels	T
Social selection only > non-social selection only					
Right cerebellum (IX)	4	−54	−44	191	6.91***
Left cerebellum (IX)	−6	−56	−44		6.06**
Left cerebellum (crus 2)	−22	−78	−36	126	5.33*
Left angular gyrus, including TPJ	−40	−56	26	737	6.65***
Left angular gyrus	−54	−64	24		5.09*
Left precuneus	−8	−52	34	2256	8.18***
Right precuneus	2	−56	34		7.35***
Left precuneus	0	−66	28		6.29**
Right supramarginal gyrus	54	−46	26	885	6.42***
Right angular gyrus	44	−58	24		5.46*
Right superior frontal gyrus	20	34	40		4.490
Left superior medial gyrus, including mPFC	2	54	24	565	5.69**
Left superior medial gyrus	−8	50	38		5.14*
Left superior medial gyrus	−10	52	30		4.780
Social selection only < non-social selection only					
Left middle temporal gyrus	−52	−54	−4	363	6.02**
Left inferior parietal lobule	−58	−36	48	768	6.26**
Left inferior parietal lobule	−46	−40	48		5.01*
Left inferior parietal lobule	−36	−44	38		3.90*
Left superior medial gyrus	0	30	48	142	4.61
L IFG p. triangularis	−48	36	22	297	5.48*

Coordinates refer to the MNI stereotaxic space. Whole-brain analysis thresholded at cluster-defining uncorrected*P* < 0.001 and cluster-wise FWE corrected *P* < 0.05, with voxel extent ≥ 10. IFG = inferior frontal gyrus.

* *P* < 0.05,

** *P* < 0.01,

*** *P* < 0.001 (peak FWE corrected).

#### Social sequencing vs social selection only

To test the hypothesis that the posterior cerebellar crus is preferentially involved in the sequencing rather than selection of social actions, we computed a social sequencing > social selection-only contrast. The results indicated no significant activations in the cerebellum; however, an ROI analysis using small volume correction (see Method section) showed activation in cerebellar crus 1. A whole-brain analysis revealed further activations in the middle temporal gyrus, superior frontal gyrus and middle frontal gyrus. The TPJ and mPFC were not activated, which is not surprising since social actions are included in the two conditions that are contrasted. The opposite contrast (social selection only > social sequencing) revealed no activation in any area of interest (i.e. crus, TPJ and mPFC).

#### Non-social sequencing vs non-social selection only

To further verify the hypothesis that the cerebellar crus is preferentially involved in sequencing social actions rather than non-social actions, we ran the same contrast for the non-social conditions, that is, non-social sequencing > non-social selection only (Table 3). None of these contrasts revealed activation in any of the predicted posterior cerebellar or cortical (mentalizing) areas for both the whole-brain and ROI analyses. The whole-brain contrast further revealed activations in the middle temporal gyrus, fusiform gyrus, posterior cingulate cortex (PCC), precentral gyrus, superior frontal gyrus, middle frontal gyrus and the superior frontal gyrus, whereas the opposite contrast revealed activations in the cuneus.

**Table 3.** Whole-brain analysis contrasting social/non-social sequencing against social/non-social selection only

	MNI coordinates				
Brain label/contrast	*x*	*y*	*z*	Voxels	*t*
Social sequencing > social selection only					
ROI: Left cerebellum (crus 1)	−30	−64	−32	60	4.21*
	−44	−58	−34		3.97*
Left middle temporal gyrus	−44	−70	8	12 811	9.23***
Right superior parietal lobule	28	−60	60		7.32***
Right middle occipital gyrus	32	−74	38		7.16***
	6	−38	24	481	5.57*
Left superior frontal gyrus	−22	4	58	811	6.10**
Left middle frontal gyrus	−26	12	56		5.67**
Left superior frontal gyrus	−22	2	72		5.33*
Right superior frontal gyrus	26	8	68	1765	6.91***
Right middle frontal gyrus	28	0	52		6.60***
Right IFG p. opercularis	50	10	26		5.31*
Left middle frontal gyrus	−24	30	42	198	6.34**
Right middle frontal gyrus	40	40	26	364	4.89*
Left IFG p. triangularis	−34	32	26	257	4.27
Social sequencing < social selection only					
Right cuneus	8	−90	22	508	4.74
Left calcarine gyrus	−6	−94	10		4.72
Right calcarine gyrus	14	−84	12		4.66
	28	−46	24	193	4.43
Non-social sequencing > non-social selection only					
Left middle temporal gyrus	−44	−70	8	6280	8.92***
Left middle occipital gyrus	−42	−70	16		7.79***
Right precuneus	2	−56	52		6.21**
Left fusiform gyrus	−28	−62	−8	140	6.06**
Right PCC	4	−40	24	117	4.85
Right precentral gyrus	30	0	50	415	4.73
Right superior frontal gyrus	20	8	64	432	6.19**
Right middle frontal gyrus	24	34	40	346	5.78**
Right superior temporal gyrus	52	−42	14	195	4.61
Non-social sequencing < non-social selection only					
Right cuneus	14	−94	22	150	4.49

Coordinates refer to the MNI stereotaxic space. Whole-brain analysis thresholded at cluster-defining uncorrected *P* < 0.001 and cluster-wise FWE corrected *P* < 0.05, with voxel extent ≥ 10. ROI analysis using a small volume correction with a sphere of 15 mm around a priori MNI coordinates (±40, −70, −40; Van Overwalle *et al.*, 2020a).

* *P* < 0.05,

** *P* < 0.01,

*** *P* < 0.001 (peaks FWE corrected).

#### Parametric analysis using confidence ratings

We further performed a parametric regression analysis using participant’s confidence ratings as a parameter. Because whole-brain activation showed only marginally significant results, we narrowed down the analysis to an ROI analysis (see Method section). This analysis revealed that the confidence ratings were positively correlated with significant activation in cerebellar crus 1 (MNI coordinates −44 −74 −32) in the social sequencing > social selection contrast (*P* = 0.03). No relationship was found in the other contrasts.

## Discussion

Prediction is an essential part of our social lives, as we continuously adapt and fine-tune our anticipations of future interactions using information inferred from the past, such as traits, which summarize what another person is like. Previous research has demonstrated the role of the cerebellar crus in inferring traits from social action sequences (Baetens *et al.*, 2014; Pu *et al.*, 2020). In this study, we reversed this logic. We investigate the novel hypothesis that the posterior cerebellar crus uses internal models to predict others’ ongoing social action sequences based on their known personality traits. We found significant cerebellar involvement when participants were asked to predict an upcoming sequence of social actions based on prior trait information.

### The posterior cerebellar crus is involved in predicting action sequences

As predicted in our hypothesis, we found activation of the posterior cerebellar crus when participants had to predict sequences of social behaviors compared to the control conditions where they had to (i) predict sequences of non-social objects or (ii) only select social material (without generating a sequencing). This indicates that social action and its associated anticipated temporal sequence are necessary to obtain activation of the posterior crus. These results support the role of the cerebellum in predicting the temporal coordination of future social actions based on prior social judgments on persons. Although using the reverse logic, this is in line with prior research demonstrating the functional role of the cerebellar crus in social sequence detection during mentalizing in recent fMRI research using social sequencing tasks such as false beliefs in the picture and verbal sequencing tasks (Heleven *et al.*, 2019), trait attributions in a sequencing memory task (Pu *et al.*, 2020), as well as in studies with cerebellar patients depicting human behavior (Leggio *et al*., 2008), and using false beliefs in the picture sequencing task (Van Overwalle *et al.*, 2019a). The novel contribution of our current study is that it extends this line of research on social inferences based on social action sequences to the reverse logic of predicting social action sequences based on personality traits. More generally, the present results support and further contribute to the ‘sequencing hypothesis’ (Leggio and Molinari, 2015), which posits that the posterior cerebellum builds internal models based on previous or ongoing interactions, to predict how other people’s actions will be executed, so that we can automatize current and future interactions, while detecting possible violations of our predictions (see also Van Overwalle *et al*., 2019b, 2020b).

The present task demonstrated that the presence of both sequencing and social elements recruited the posterior cerebellum. This stands in contrast to previous research that involved social mentalizing without clear temporal sequencing and showed inconsistent results: either finding cerebellar activation (Todorov *et al.*, 2007; Baetens *et al.*, 2014; Van Overwalle *et al.*, 2016) or failing to show cerebellar activation (Sokolovsky *et al.*, 2010; Hoche *et al.*, 2016). One explanation is that while these studies included social elements, they did not reveal cerebellar activation because they did not include clear sequencing elements. Note, however, that although our hypothesized effects were often significant in a whole-brain analysis, in some contrasts, cerebellar activation was only significant when testing effects within hypothesized ROIs (thus limiting the required corrections for multiple comparisons). Prior research might have underestimated the role of the posterior cerebellum, because researchers did not look for it in a similar hypothesis-driven way.

It should be noted that our results (Figure 2) also reveal significant activation in the posterior crus during selection only (without sequencing), although those activations are weaker than those for sequencing. There are several potential explanations for this. First, the posterior crus is possibly receptive to social information in general, but even more so when explicit sequencing is involved. Second, a related explanation is that there is some specialization in the posterior crus depending on whether human social actions include sequencing or not. A recent meta-analysis (Van Overwalle *et al.*, 2020b) found that the pre-posterior crus (termed ‘sequencing’; MRI coordinates ±24 −76 −40) preferentially processes sequences contained in social information, while the ulterior posterior crus (derived from the NeuroSynth database; MRI ±26 −84 −34) is relatively more receptive to social information in general. Third, it is also possible that the cerebellar crus responds not only to explicit but also to implicit sequencing, due to imagination, stereotyping or automatization of highly trained action sequences. In support of this, a recent study found that the cerebellar crus was recruited during training and the subsequent execution of implicit true–false belief orientation sequences (Ma *et al.*, 2021). Fourth, the cerebellar crus might be activated even without explicit sequencing due to cerebello-cortical closed loops which automatically exchange information between mentalizing cortical areas and the posterior cerebellum to check and predict previously learned and automatized action sequences. This is supported by effective connectivity research showing that these loops appear to always be functionally active regardless of whether social or non-social information is processed (Van Overwalle, Van de Steen, *et al.*, 2019, 2020c).

Interestingly, this study highlights possible social cognitive functions of the cerebellar lobule IX which are hereto largely unknown, although this area also belongs to the default/mentalizing network (Buckner *et al.*, 2011). Our results demonstrated that predicting sequences of social behaviors revealed activation of the cerebellar lobule IX. Additionally, D’Mello *et al.* (2015) found that gray matter reductions in cerebellar lobule IX, alongside reductions in the cerebellar crus 1 and crus 2, were associated with an increase in social impairments, in particular, in social interactions. Our results highlight the potential preferential role of cerebellar lobule IX in social prediction. Given that this area is located in the anterior cerebellum specialized for motor behavior, it could suggest that social prediction might also entail an element of anticipation of movement during future social interactions. This is a suggestion that needs to be corroborated by future research.

### The posterior cerebellum and metacognition

On a daily basis, we evaluate our performance or perceived confidence on everyday tasks, even without an objective standard. Therefore, in addition to exploring the functional role of the cerebellum in prediction of social action sequences, we also wanted to explore activations during meta-cognition, that is, while participants were rating their performance confidence. Interestingly, we found that self-confidence was correlated with activation in the posterior cerebellar crus 1 for social sequencing compared to the non-social sequencing control (however, only for ROI analysis, whole-brain analysis showed no activation). This is consistent with findings of Pu *et al.* (2020), who also found correlation with activation of the cerebellar crus 1 and confidence ratings when participants were asked to rate their retrieval confidence on a sequence task. This might point to a specialized role of the cerebellar crus 1 in meta-cognition during mentalizing.

### Frontal and parietal brain regions and retrieving social sequences

Along with robust activations of the cerebellum in the social domain, we also found robust activation in the cerebral cortex under the same conditions. Specifically, we found activation of the TPJ and the mPFC in the social sequencing condition, cortical areas which are linked to the mentalizing network (Van Overwalle and Baetens, 2009; Schurz *et al.*, 2014). This activation was not observed in any of the non-social conditions. This is consistent with our prediction that these key mentalizing areas would be activated, together with the cerebellar key mentalizing areas crus 1 and crus 2. Activations of mentalizing areas further support our hypothesis that posterior cerebellar activations in our study reflect mentalizing capacity during prediction of other’s social action sequences.

### Limitations and questions

Our results suggest stronger cerebellar activation for social sequencing as opposed to non-social sequencing. A potential limitation is that this effect results from the increased difficulty of the social sequencing material as opposed to the non-social material. However, this is unlikely, as participants’ accuracy and reaction times on these tasks were comparable.

Our results suggest a stronger cerebellar activation for sequencing as opposed to non-sequencing in the selection-only task. A potential limitation is that participants in the selection-only task can in principle respond by judging only the third sentence (following the two neutral sentences), because they may have quickly learned that the first two sentences are not relevant for judging trait consistency. If so, this may have rendered the selection-only task less difficult, resulting in less cerebellar crus activation.

More generally, one could argue that the use of verbal material in this experiment caused the activation of the cerebellar crus rather than its social content. This is unlikely, however. Although the cerebellar crus is involved in both mentalizing and language, one could argue that it is actually the inclusion of social content in language studies (Frings *et al.*, 2006; D’Mello *et al.*, 2017) that accounts for cerebellar crus activation and not the other way around. When looking at the material in many language studies, it is evident that sentences typically involve social agents, actions or social interactions, or at least imply them, an element that is often overlooked and not controlled for in language research. For example, Frings *et al.* (2006) conducted a study where participants had to provide a verb to a corresponding noun, such as ‘drive’ for the noun ‘car’, which implies the social action that an agent ‘drives’ the ‘car’. As another example, D’Mello *et al.* (2017) investigated the predictability of sentences such as ‘the man looked at …’ which clearly reveal a social action. Moreover, the cartoon-like picture sequencing task mentioned earlier does not involve any linguistic elements but nonetheless shows comparable fMRI activation in the cerebellum as for the verbal sequencing version (Heleven *et al.*, 2019). More generally, a recent meta-analysis by Van Overwalle *et al.* (2020b) mentioned earlier documented that less than 10% of the studies that activated crus 2 involved pure semantics without any social content, so that language per se might constitute only a minor factor. Furthermore, it has been suggested that communication and language primarily serve a social purpose (for review, see Li and Jeong, 2020) and that language developed in synchrony with social group size and social behavior during human evolution (e.g. Pinker, 2010; Dunbar, 2017). For all these reasons, language cannot easily be disentangled from sociality. Although we prefer a social explanation for the present results, we cannot entirely rule out that language played an additional role in the present results.

Recall that in our study, there were always two persons involved in an interaction. One might therefore argue that the inclusion of a social solo (non-interactive) sequencing control could have been helpful to verify if (and to what extent) activation is due to the inclusion of a social agent, as opposed to social interactions. In this way, we could have had a stronger argument for the involvement of the posterior cerebellum in predictions of social action sequences. The distinction between activations in solo-social and social interactive contexts, however, could be an interesting addition for future research.

## Conclusion

The present study investigated the role of the posterior cerebellar crus in social action prediction based on trait information. In line with our hypothesis guided by the ‘sequencing hypothesis’ (Van Overwalle *et al.*, 2019b, 2020b), our findings highlight the crucial role of the posterior cerebellar crus 1 and crus 2, along with that of the cerebellar lobule IX, in the prediction of social event sequences.
